# *Culicoides* (*Avaritia*) *gornostaevae* Mirzaeva, 1984 (Diptera: Ceratopogonidae) - a possible vector species of the Obsoletus group new to the European fauna

**DOI:** 10.1186/1756-3305-7-445

**Published:** 2014-09-30

**Authors:** Carsten Kirkeby, Patrycja Dominiak

**Affiliations:** National Veterinary Institute, Technical University of Denmark, Bülowsvej 27, DK-1870 Frederiksberg C, Denmark; Department of Invertebrate Zoology and Parasitology, University of Gdańsk, Wita Stwosza 59, 80-308 Gdańsk, Poland

**Keywords:** Diptera, Ceratopogonidae, *Culicoides*, Obsoletus group, New records, Europe

## Abstract

**Background:**

*Culicoides gornostaevae* Mirzaeva, 1984, known previously only from Siberia, is a boreal species included into the Obsoletus group of *Culicoides* sg. *Avaritia*. Members of the subgenus can act as vectors of various diseases. In Europe they are involved in the transmission of the Schmallenberg virus and bluetongue virus.

**Findings:**

*Culicoides gornostaevae* Mirzaeva, 1984 is reported for the first time in Europe with new country records from Norway, Poland and Sweden.

**Conclusions:**

*Culicoides gornostaevae* Mirzaeva, 1984 has not been previously mentioned from Europe, even though there has been an extensive monitoring of *Culicoides* species during the last decades. Most probably this species has been notoriously overlooked in the materials, because of the problems with identification of the females of the subgenus *Avaritia*. Similar to other species of the Obsoletus group, *C. gornostaevae* should be regarded as a possible vector for Schmallenberg and bluetongue virus.

## Findings

### Background

There are over 1300 extant species of *Culicoides* in the world fauna [1], but only approximately 30 are known to act as vectors for diseases [2]. Fourteen of these vector species are placed in the subgenus *Avaritia*[2], and among them are also some *Culicoides* of the Obsoletus group. Three of the six species included into this species group [3] have been previously recorded in Europe: *C. montanus* Shakirzjanova, 1962, *C. obsoletus* (Meigen, 1818) and *C. scoticus* Downes & Kettle, 1952. However, males of *C. montanus* have never been collected and observed [3]. The remaining three species of the Obsoletus group are *C. gornostaevae* Mirzaeva, 1984 and *C. sinanoensis* Tokunaga, 1937, which have been recorded from Russia and eastwards to Japan [4], and a North American species *C. sanguisuga* (Coquillett, 1901). Since the outbreak of bluetongue virus in northern Europe in 2006, a lot of research has been focused on the females of *Culicoides* midges and on their role as vectors. Males in the Obsoletus group are easy to distinguish by the shape of the genitalia, but the females are notoriously difficult to separate by morphology. Therefore, molecular analyses have been conducted to identify specimens to the species level, but it is often not possible to examine the whole amount of materials in large monitoring programmes for vectors. Thus, it is probable that in some cases female specimens of this group could have been misidentified, especially if the species considered have been chosen by geography only. Moreover, it is noteworthy that an intense monitoring of potential vector species during the last decades has mostly focused on vectors near farms, hence the potential vector species in other habitats that affect wildlife species such as roe deer and red deer may have been overlooked [5, 6].

We here report findings of *Culicoides gornostaevae* from Norway, Poland and Sweden for the first time.

The division into the zoogeographic regions follows Holt *et al*. [7].

### Systematics

#### Culicoides gornostaevae Mirzaeva

*Culicoides sanguisuga*: Gornostaeva 1977 [8]: 493 (Russia). Nec *C. sanguisuga* (Coquillet, 1901).

*Culicoides gornostaevae* Mirzaeva, 1984 [9]: 371 (Russia).

*Culicoides* (*Avaritia*) *gornostaevae*: Mirzaeva 1989 [10]: 62 (Russia); Glukhova 1989 [4]: 187 (Russia); Mirzaeva 2000 [11]: 422 (Russia); Glukhova 2005 [12]: 12, 17 (in key).

*Culicoides obsoletus*: Hagan *et al*. 2000 [13]: 469 (Norway). Nec *C. obsoletus* (Meigen, 1818).

### New country records

**Norway SW.** Geitaknottane, Kvam municipality in Hordaland County (60.05°N 5.53°E, 180-200 m.a.s.l.), coastal forest, 28 May 1998, fogging, 1 male, leg. J. Skartveit & K.H. Thunes. **Poland N.** Brzyno n. Wejherowo, umbelliferae flowers, 2 June 1982, 1 male, leg. R. Szadziewski. Gdańsk, zoological garden, 1 June 1979, at light, 1 male. **Poland NE.** Silec n. Kętrzyn, 8 June 1980, near forest margin, swarming, 7 males, leg. R. Szadziewski. **Poland S.** Orava-Nowy Targ Basin, Czarny Dunajec Baligówka, umbelliferae flowers, 27 June 2006, net, 2 males, leg. P. Dominiak. **Poland SE.** Ustrzyki Górne, 29 July 1980, swarming, 1 male, leg. R. Szadziewski. **Sweden S.** Vivljunga n. Markaryd (56.58501°N 13.48143°E), 7 June 2014, swarming, 3 males, leg. C. Kirkeby. These specimens were caught in a mixed swarm with *C. impunctatus* females.

### Distribution and feeding habits

**Figure 1:**
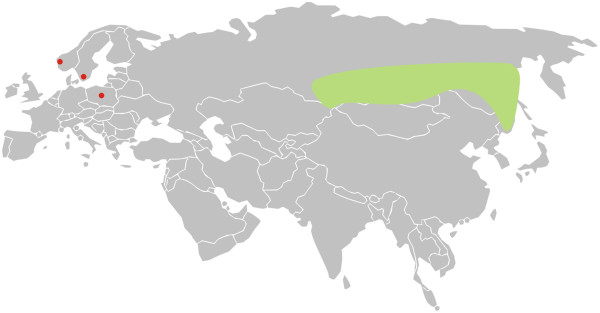
**Map of the distribution patterns of** ***Culicoides gornostaevae*** **Mirzaeva.** New country records marked in red, the former ones from Siberia are shown in green.

*Culicoides gornostaevae* is a boreal Palaearctic species, widely distributed in the western and middle part of Siberia, between the Uba River and the Amur River (Figure 1). According to data from the literature it is especially numerous in the forest zone of Altai, Khakassia, Gornaya Shoria and Western Sayan. However, it is important to indicate that almost all of the former records are based on females only. This biting midge species is new for the fauna of Norway, Poland and Sweden. Females of *C. gornostaevae* are known to attack humans and other animals [4].

### Morphology

**Figure 2:**
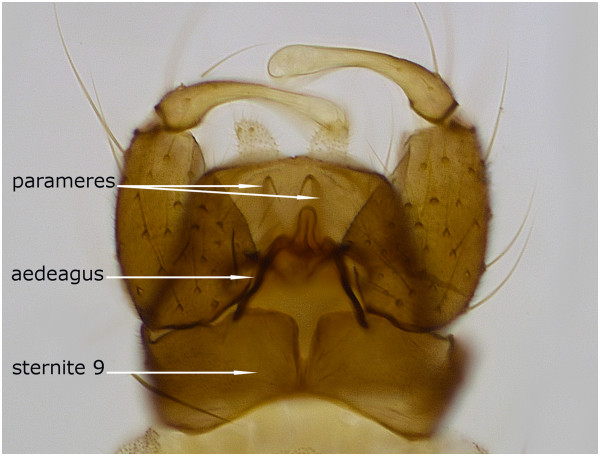
**Male genitalia of** ***Culicoides gornostaevae*** **Mirzaeva, ventral view.** Specimen from Poland.

**Figure 3:**
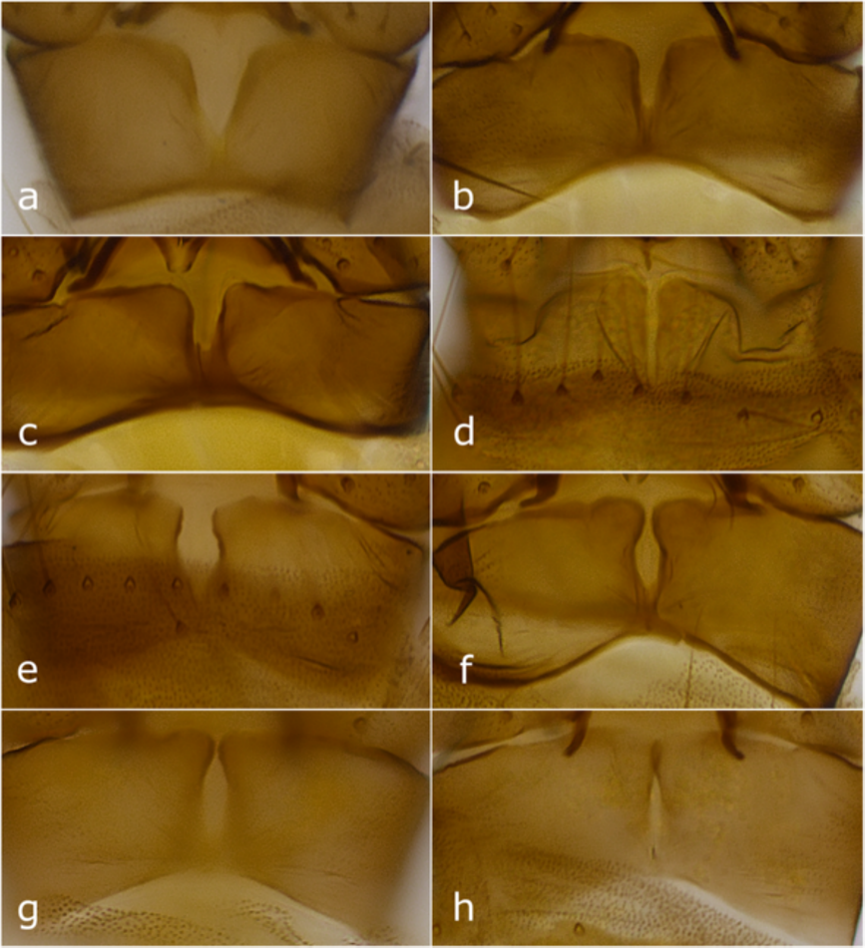
**Sternite 9 in male genitalia of** ***Culicoides*** ***gornostaevae*** **Mirzaeva (a-d) and** ***C. obsoletus*** **(Meigen) (e-h), ventral view.** Specimens of *C. gornostaevae* from: a - Sweden; b, c - Poland; d - Norway.

**Figure 4:**
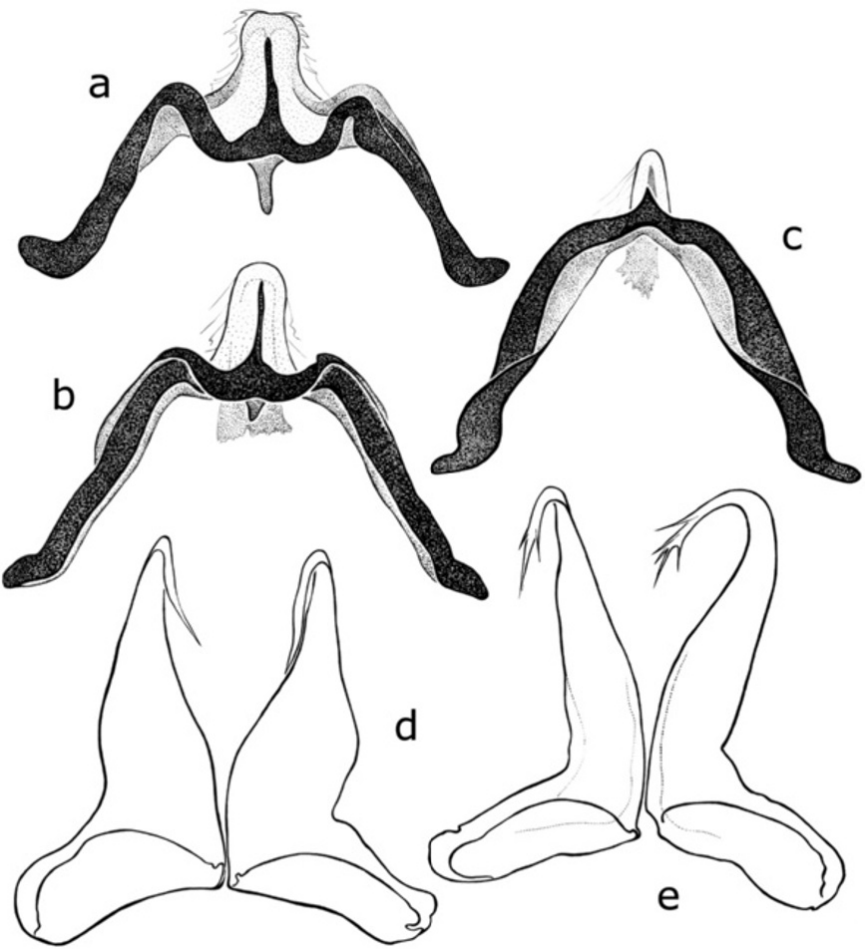
**Aedeagus (a-c) and parameres (d, e) in male genitalia of** ***Culicoides gornostaevae*** **Mirzaeva (a, b, d) and** ***C. obsoletus*** **(Meigen) (c, e).** Specimens of *C. gornostaevae* from: a-Norway, b-Poland, d-Poland.

*Culicoides gornostaevae* closely resembles *C. obsoletus*, but these two species show clear differences in male genitalia armature. Males of *C. gornostaevae* (Figure 2) are characterized by widely opened triangular or nearly slit-like excavation of the distal margin of the 9th sternite (Figure 3a-d), and by bare tips of parameres (Figure 4d). The cleft of the 9th sternite in *C. obsoletus* is oval with distinctly converging distal edges (Figure 3e-h), while tips of parameres are covered with short but prominent hair (Figure 4e). The shape of aedeagus is similar in both species and often depends on different orientation of specimens after mounting them on a microscope slide. However, apicolateral parts of the aedeagal arch seems to be rather angular or trapezoidal in *C. gornostaevae* (Figure 4a,b) and more rounded in *C. obsoletus* (Figure 4c). A transparent membrane attached to the apical projection of aedeagus (sometimes referred to as spines or teeth), which is usually mentioned as a characteristic feature for male of *C. gornostaevae* only [4, 8–10, 12], even if sometimes not clearly visible, is always present in both species. We do not find distribution and intensity of wing patterns as useful in morphological identification of the species of the Obsoletus group, at least in males. These characters are highly variable, and among material examined both specimens with well visible wing patterns as well as those with very pale, indistinct patterns were present.

Although females of the subgenus *Avaritia* have nearly no diagnostic features, and thus are easily misidentified, the descriptions of some species are based only on this sex. Among them are *Culicoides sanguisuga* known from the Nearctic and *C. montanus*, originally described from montane regions in Kazakhstan but reported from both the Palaearctic and the Saharo-Arabian regions. The first detailed illustrations of males of these two species can be found in Jamnback & Wirth [14] (or in [15]) and in Gutsevich [16] (the same drawing was subsequently used in [9, 10, 17]) respectively. It is probable that the names *C. gornostaevae* and *C. sanguisuga*, and maybe also *C. montanus*, should be treated as synonyms, but further studies, including a molecular analysis, and examination of more materials are necessary to confirm this supposition. Male genitalia of the two first mentioned species look very much alike, but according to Mirzaeva’s [9] description of *C. gornostaevae*, wings in this species are a little bit darker and have less prominent color patterns in comparison to those in *C. sanguisuga*. The diagnostic characters given by Gutsevich [16] for male *C. montanus* are based probably on a single specimen. According to this paper (ibidem) the sensory pit on 3rd palpal segment is rather deep. The aedeagal arch in male genitalia of this species seems to be quite slender (similar to this in *C. obsoletus*), but the shape of the cleft in sternite 9 as well as bare tips of parameres indicate that *C. montanus* is very close to *C. gornostaevae*. Female of *C. montanus* is characterized by a stout 3rd palpal segment bearing a deep sensory pit [4, 9, 10, 16–18] and by the presence of peg-like sensilla on proximal flagellomeres [4, 9, 10, 16, 17].

## Discussion

Delimitation of the species within the Obsoletus group still remains problematic, and females are especially difficult to distinguish by morphology [19]. There are no proper diagnostic characters for female of *Culicoides gornostaevae*, therefore, this species may have been identified as *C. obsoletus* and overlooked in trap catches. Furthermore, the males are usually not caught in large numbers in light traps, and it could be the explanation why this species has not been reported in any previous studies concerned with the *Culicoides* midges in Europe. The present records are based on males collected between 1979 and 2014, which indicates that this species is well established here. Because many of the *Culicoides* species of the subgenus *Avaritia* are known to transmit various diseases, and due to its close relationship to *C. scoticus* and *C. obsoletus*, *C. gornostaevae* should also be regarded as a potential vector of bluetongue virus or Schmallenberg virus in Europe.

## Conclusion

We here report the first records of *Culicoides gornostaevae* from Norway, Poland and Sweden. This species is morphologically close to *C. obsoletus* but these both species differ in the armature of the male genitalia. *Culicoides gornostaevae* belongs to the subgenus *Culicoides* (*Avaritia*), and similarly to *C. scoticus* and *C. obsoletus*, should be considered as possible vector species of bluetongue virus and Schmallenberg virus in Europe.
